# Supplementary material for: The motivation to inform others: a field experiment with wild chimpanzees
Source: PeerJ. 2024 Dec 6;12:e18498. doi: 10.7717/peerj.18498 (PMC11627086; doi:10.7717/peerj.18498)
Supplement: Supplemental Information 3 [file peerj-12-18498-s003.docx]

**SUPPLEMENTARY INFORMATION**

| **Table S1. Startle responses observed across conditions.** | | | | | | | |
| --- | --- | --- | --- | --- | --- | --- | --- |
| **ID** | **Trial Number** | **Condition** | | **Startle** | |  |  |
| **ZG** | 1 | Non-Social | | 0 | |  |  |
| **SQ** | 1 | Non-Social | | 0 | |  |  |
| **KT** | 1 | Social | | 0 | |  |  |
| **FK** | 1 | Social | | 0 | |  |  |
| **MS** | 1 | Social | | 1 | |  |  |
| **ZF** | 1 | Social | | 0 | |  |  |
| **KZ** | 1 | Non-Social | | 0 | |  |  |
| **PS** | 1 | Non-Social | | 0 | |  |  |
| **ZL** | 1 | Social | | 0 | |  |  |
| **ZG** | 2 | Non-Social | | 1 | |  |  |
| **KT** | 2 | Social | | 0 | |  |  |
| **FK** | 2 | Social | | 0 | |  |  |
| **SQ** | 2 | Non-Social | | 0 | |  |  |
| **MS** | 2 | Social | | 0 | |  |  |
| **KZ** | 2 | Non-Social | | 0 | |  |  |
| **PS** | 2 | Non-Social | | 0 | |  |  |
| **ZF** | 2 | Social | | 1 | |  |  |
| **ZL** | 2 | Social | | 0 | |  |  |
| **Table S2. Raw number of alarm calls observed per individual in each trial of each condition** | | | | | | | |
|  | |  | | **Trial** | |  | |
| **ID** | | **Condition** | | **1** | | **2** | |
| **Kwezi** | | Non-social | | 0 | | 0 | |
| **Pascal** | | Non-social | | 3 | | 0 | |
| **Squibs** | | Non-social | | 1 | | 0 | |
| **Zig** | | Non-social | | 1 | | 2 | |
| **Frank** | | Social | | 4 | | 0 | |
| **Kato** | | Social | | 0 | | 1 | |
| **Musa** | | Social | | 0 | | 0 | |
| **Zefa** | | Social | | 5 | | 3 | |
| **Zalu** | | Social | | 0 | | 1 | |

| **Table S3. Total looking duration (s) towards the predator model observed per individual in each trial of each condition** | | | |
| --- | --- | --- | --- |
|  |  | **Trial** |  |
| **ID** | **Condition** | **1** | **2** |
| **Kwezi** | Non-social | 26.34 | 10.84 |
| **Pascal** | Non-social | 6.65 | 12.70 |
| **Squibs** | Non-social | 18.00 | 7.62 |
| **Zig** | Non-social | 24.74 | 11.80 |
| **Frank** | Social | 30.00 | 21.24 |
| **Kato** | Social | 11.65 | 18.00 |
| **Musa** | Social | 23.08 | 30 |
| **Zefa** | Social | 17.37 | 16.85 |
| **Zalu** | Social | 29.12 | 14.71 |
